# Quantification of mitral regurgitation in patients with hypertrophic cardiomyopathy using aortic and pulmonary flow data: impacts of left ventricular outflow tract obstruction and different left ventricular segmentation methods

**DOI:** 10.1186/s12968-017-0417-8

**Published:** 2017-12-21

**Authors:** Mateusz Śpiewak, Mariusz Kłopotowski, Monika Gawor, Agata Kubik, Ewa Kowalik, Barbara Miłosz-Wieczorek, Maciej Dąbrowski, Konrad Werys, Łukasz Mazurkiewicz, Katarzyna Kożuch, Magdalena Polańska-Skrzypczyk, Joanna Petryka-Mazurkiewicz, Anna Klisiewicz, Zofia T. Bilińska, Jacek Grzybowski, Adam Witkowski, Magdalena Marczak

**Affiliations:** 1 Magnetic Resonance Unit, Department of Radiology, Institute of Cardiology, Warsaw, Poland; 2 Department of Interventional Cardiology and Angiology, Institute of Cardiology, Warsaw, Poland; 3 Department of Cardiomyopathy, Institute of Cardiology, Warsaw, Poland; 4 Department of Congenital Heart Diseases, Institute of Cardiology, Warsaw, Poland; 5 Oxford Centre for Clinical Magnetic Resonance Research, John Radcliffe Hospital, Headington, Oxford, UK; 6 Department of Coronary and Structural Heart Diseases, Institute of Cardiology, Warsaw, Poland; 7 Unit for Screening Studies in Inherited Cardiovascular Diseases, Institute of Cardiology, Warsaw, Poland

**Keywords:** Hypertrophic cardiomyopathy, Mitral regurgitation, Phase-contrast, Cardiovascular magnetic resonance, Left ventricular outflow tract obstruction

## Abstract

**Background:**

Cardiovascular magnetic resonance (CMR) imaging in patients with hypertrophic cardiomyopathy (HCM) enables the assessment of not only left ventricular (LV) hypertrophy and scarring but also the severity of mitral regurgitation. CMR assessment of mitral regurgitation is primarily based on the difference between LV stroke volume (LVSV) and aortic forward flow (Ao) measured using the phase-contrast (PC) technique. However, LV outflow tract (LVOT) obstruction causing turbulent, non-laminar flow in the ascending aorta may impact the accuracy of aortic flow quantification, leading to false conclusions regarding mitral regurgitation severity. Thus, we decided to quantify mitral regurgitation in patients with HCM using Ao or, alternatively, main pulmonary artery forward flow (MPA) for mitral regurgitation volume (MRvol) calculations.

**Methods:**

The analysis included 143 prospectively recruited subjects with HCM and 15 controls. MRvol was calculated as the difference between LVSV computed with either the inclusion (LVSV_incl_) or exclusion (LVSV_excl_) of papillary muscles and trabeculations from the blood pool and either Ao (MRvol_Aoi_ or MRvol_Aoe_) or MPA (MRvol_MPAi_ or MRvol_MPAe_). The presence or absence of LVOT obstruction was determined based on Doppler echocardiography findings.

**Results:**

MRvol_Aoi_ was higher than MRvol_MPAi_ in HCM patients with LVOT obstruction [47.0 ml, interquartile range (IQR) = 31.5–60.0 vs. 35.5 ml, IQR = 26.0–51.0; *p* < 0.0001] but not in non-obstructive HCM patients (23.0 ml, IQR = 16.0–32.0 vs. 24.0 ml, IQR = 15.3–32.0; *p* = 0.26) or controls (18.0 ml, IQR = 14.3–21.8 vs. 20.0 ml, IQR = 14.3–22.0; *p* = 0.89). In contrast to controls and HCM patients without LVOT obstruction, in HCM patients with LVOT obstruction, aortic flow-based MRvol (MRvol_Aoi_) was higher than pulmonary-based findings (MRvol_MPAi_) (bias = 9.5 ml; limits of agreement: −11.7–30.7 with a difference of 47 ml in the extreme case). The differences between aortic-based and pulmonary-based MRvol values calculated using LVSV_excl_ mirrored those derived using LVSV_incl_. However, MRvol values calculated using LVSV_excl_ were lower in all the groups analyzed (HCM with LVOT obstruction, HCM without LVOT obstruction, and controls) and with all methods of MRvol quantification used (*p* ≤ 0.0001 for all comparisons).

**Conclusions:**

In HCM patients, LVOT obstruction significantly affects the estimation of aortic flow, leading to its underestimation and, consequently, to higher MRvol values than those obtained with MPA-based MRvol calculations.

**Electronic supplementary material:**

The online version of this article (doi:10.1186/s12968-017-0417-8) contains supplementary material, which is available to authorized users.

## Background

Up to 70% of patients with hypertrophic cardiomyopathy (HCM) exhibit left ventricular (LV) outflow tract (LVOT) obstruction [1]. LVOT obstruction is frequently associated with significant mitral regurgitation as a result of systolic anterior motion of mitral leaflets [2]. Less commonly, mitral regurgitation is related to intrinsic valve abnormalities [2]. LVOT obstruction together with systolic anterior motion-related mitral regurgitation leads to progressive deterioration of clinical status. Significant symptomatic LVOT obstruction requires septal reduction therapy, which may vary depending on the mechanisms of LVOT obstruction, mitral valve disease status and alterations in papillary muscles [3, 4]. The coexistence of mitral regurgitation may influence the decision between intervention and conservative treatment, as some centers will consider septal reduction therapy in HCM patients with mild symptoms and significant LVOT obstruction provided that they have moderate-to-severe systolic anterior motion related mitral regurgitation [3]. The mechanism of mitral regurgitation is crucial for deciding between alcohol septal ablation and surgical myectomy since intrinsic mitral valve abnormalities leading to regurgitation can be adequately addressed only by surgery. Additionally, the extent of surgery (myectomy alone or myectomy with adjunctive procedures ranging from anterior mitral leaflet plication to mitral valve replacement) depends on, among other things, the severity of mitral regurgitation [2–4]. Lastly, mitral regurgitation is one of the factors responsible for left atrial enlargement and is an important cause of atrial arrhythmias, particularly atrial fibrillation [3].

Over the past decade, cardiovascular magnetic resonance (CMR) imaging has emerged as a valuable tool for the non-invasive assessment of patients with HCM [5, 6]. CMR imaging provides prognostic information and valuable data on cardiac anatomy, mechanisms of LVOT obstruction, and differential diagnoses of LV hypertrophy [3, 5, 7–9]. CMR imaging has also been shown to be an accurate and reproducible method of flow quantification, including valve regurgitation grading [8, 10–13]. In the case of the mitral valve, the mitral regurgitant volume (MRvol) is usually quantified as the difference between LV stroke volume (LVSV) – measured by volumetric assessment – and aortic forward flow (Ao) – measured by phase-contrast (PC) velocity mapping [12]. However, several factors have been shown to limit the accuracy of the PC technique, including complex flow patterns, as shown in patients with bicuspid aortic valves and aortic valve stenosis [14, 15]. Similarly, LVOT obstruction in patients with HCM also leads to turbulent flow and flow measurement errors; therefore, it may have important consequences for the calculation of MRvol. These issues, however, have not yet been adequately addressed. We hypothesized that LVOT obstruction causing turbulent, non-laminar flow in the ascending aorta, leads to alterations in Ao measurements and, hence, impacts MRvol quantification in HCM patients with LVOT obstruction. In patients without intra- or extracardiac shunts, net aortic flow is almost equal to pulmonary net flow, and the latter may alternatively be used for the calculation of mitral regurgitation severity [16]. To verify this hypothesis, we measured MRvol using both aortic and pulmonary flow determined by CMR imaging in patients with HCM with and without LVOT obstruction.

## Methods

### Study population

Consecutive patients referred for CMR imaging who had a confirmed diagnosis of HCM or were suspected of having HCM were prospectively recruited from the beginning of January 2015 through the end of January 2017 at a high-volume hospital that serves as a tertiary referral center for HCM patients. Patients with irregular heart rhythm (including atrial fibrillation and frequent premature ventricular or supraventricular contractions) or a history of any septal reduction therapy were excluded. The study was approved by the local ethics committee. All patients or guardians of patients younger than 18 years of age provided written informed consent.

### CMR studies

All CMR studies were performed with a 1.5 T scanner (Avanto for 2015 examinations or Avanto^fit^ for 2016 and 2017 examinations; Siemens Healthineers, Erlangen, Germany). The imaging protocol included a stack of short-axis cine (breath-hold electrocardiogram-triggered balanced steady-state free precession) images in addition to LV long-axis cine images (typical parameters: 25 phases, echo time 1.2 ms, effective repetition time 33–54 ms, echo spacing 2.7 ms, flip angle 64–79°, slice thickness 8 mm, and gap 2 mm). Additionally, breath-hold PC velocity mapping was performed in the ascending aorta (at the level of the sinotubular junction) and the main pulmonary artery (located at the midpoint of the blood vessel), providing 30 phase and magnitude images per cardiac cycle (typical parameters: echo time 2.5 ms, effective repetition time 30–47 ms, flip angle 30°, and section thickness 5 mm). Velocity encoding sensitivity was adjusted to avoid aliasing. Imaging planes were planned perpendicular to the vessel wall based on paired orthogonal long-axis cine steady state free precession images through the LVOT or main pulmonary artery. To exclude the possible influence of physiologic factors (e.g., heart rate variability) on the difference between aortic and pulmonary flow data, PC images for Ao and main pulmonary artery (MPA) were subsequently registered one after another. To maximize gradient fidelity, particular care was taken to ensure that the vessel of interest was at the scanner’s isocenter [17, 18].

### Image analysis

**Fig. 1:**
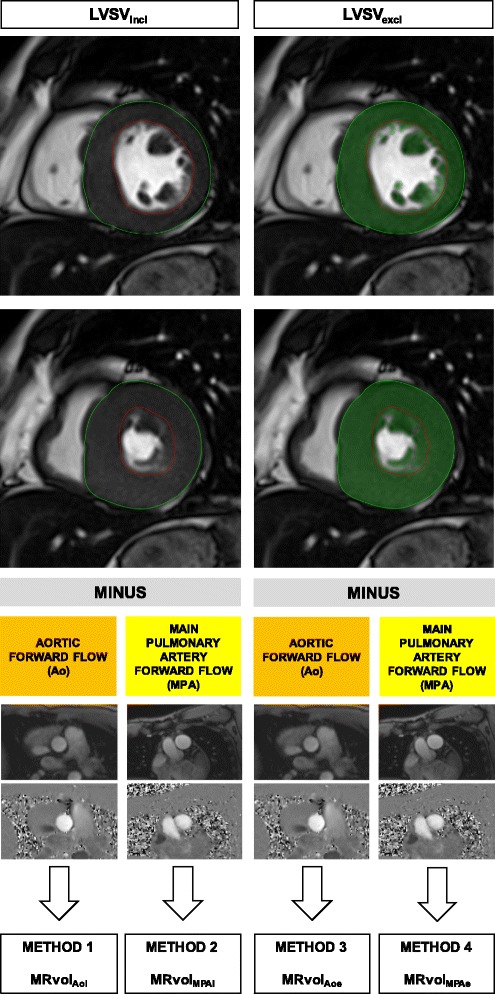
Schematic illustration of the four different methods of MRvol quantification used. Representative images of two left ventricular segmentation methods (_incl_ vs. _excl_) (top). End-diastole and end-systole are shown. *Top*, *left*: Papillary muscles and trabeculations were included (_incl_) in the blood pool, while they were excluded from the ventricular mass calculations. *Top*, *right*: Papillary muscles and trabeculations were excluded (_excl_) from the blood pool, with mass calculations performed separately for ventricular walls and papillary muscles/trabeculations

PC data were analyzed with a semiautomatic vessel edge-detection algorithm with operator correction (Argus, Siemens Healthineers). Additionally, to confirm that background phase errors did not significantly affect the results, we used dedicated software (QFlow 5.6, Medis, Leiden, the Netherlands) to perform corrections on a sample of the study cohort [all individuals in the control group and HCM group with a calculated ratio of net pulmonary flow to net aortic flow (Qp:Qs) equal to or greater than 1.2]. Aortic and pulmonary net flow was defined as the difference between forward flow and reverse flow. Ventricular volumes and mass were calculated on the basis of a stack of short-axis images from the base to the apex using dedicated software (QMass 7.6, Medis, Leiden, the Netherlands) with manual delineation of endocardial and epicardial contours in end-diastole and end-systole. LVSV was calculated as the difference between LV end-diastolic volume (LVEDV) and LV end-systolic volume (LVESV). All analyses were performed by an experienced operator (with a Level 3 Certificate from the European Association of Cardiovascular Imaging, more than 8 years of experience in CMR imaging, and experience in analyzing more than 500 CMR studies of patients with HCM). Ventricular segmentation was performed in a blinded fashion with respect to PC flow measurements (and vice versa) and patient characteristics. Two different volumetric measurement methods were used. The first method included papillary muscles and trabeculations in the blood pool (_incl_) while excluding them from the ventricular mass calculations (Fig. 1). The second method used a semiautomatic algorithm (MassK mode, QMass 7.6, Medis) enabling the exclusion of papillary muscles and trabeculations (_excl_) from the blood pool and adding them to the mass calculations (Fig. 1). The same endocardial and epicardial contours were used for both analyses.

MRvol was quantified using the following methods (MPA denotes main pulmonary artery forward flow) (Fig. 1):

- Method 1: MRvol_Aoi_ = LVSV_incl_ − Ao
- Method 2: MRvol_MPAi_ = LVSV_incl_ − MPA
- Method 3: MRvol_Aoe_ = LVSV_excl_ − Ao
- Method 4: MRvol_MPAe_ = LVSV_excl_ − MPA

### Echocardiography

All standard-of-care transthoracic echocardiography studies were performed using commercially available systems by physicians experienced in assessing patients with HCM. No patient had any invasive procedure or change in medical therapy between the CMR and echocardiography studies (median time between these studies was 34 days). As part of the routine ultrasound evaluation of patients with HCM, the presence of an LVOT gradient at rest and during provocation was assessed, with a peak gradient of 30 mmHg or higher indicating the presence of LVOT obstruction [3].

### Statistical analysis

Categorical data are presented as percentage frequencies and were analyzed using the chi-square or Fisher exact test. Continuous data are presented as the means ± standard deviation (SD) or the medians with the interquartile range (IQR). The Kolmogorov-Smirnov test was used to determine whether parameters were normally distributed. Correlations between variables with Gaussian distributions were tested using Pearson’s test. The concordance correlation coefficient, which is a measure of the agreement (both precision and accuracy) between two variables, was used for comparisons of net aortic and pulmonary flow. The Kruskal-Wallis test was used to compare continuous parameters among patients with LVOT obstruction, those without LVOT obstruction, and controls. The Bonferroni correction was applied when post hoc multiple comparisons were performed, and a *p*-value < 0.0167 (0.05÷3) was considered to indicate a significant difference. Differences between two independent groups with non-normally distributed data were assessed using the Mann-Whitney test or using the Student t-test for independent samples for data with a normal distribution. The differences between MRvol values that were estimated with various quantification methods within each study group were initially assessed using the Friedman test, while subsequent pairwise comparisons were assessed using the Wilcoxon test, with a Bonferroni-corrected *p*-value < 0.0083 (0.05 ÷ 6) denoting statistical significance. Additionally, the agreement between methods was determined by a Bland-Altman analysis demonstrating bias and 95% limits of agreement. To assess the impacts of different quantification methods on mitral regurgitation grading, we used five grades based on the calculated MRvol (< 15 ml, 15–29 ml, 30–44 ml, 45–59 ml, and ≥ 60 ml). Subsequently, we applied the kappa statistic to compare the agreement between different methods of mitral regurgitation grading within groups with different PC data (aortic vs. pulmonary). Taking into account the lack of uniform CMR thresholds for mitral regurgitation severity and the changing thresholds for mitral regurgitation grading (the most recent focused update of the American guidelines introduced a new threshold of ≥ 60 ml for severe secondary mitral regurgitation, which replaced the previous threshold of ≥ 30 ml in all other guidelines and recommendations), we intentionally used a variety of thresholds to show not only differences in binary data (severe vs. non-severe mitral regurgitation) but also more subtle differences across the spectrum of MRvol values that vary with the different quantification methods that were used [19–24]. The intraclass correlation coefficient (ICC) was used to assess the reproducibility (absolute agreement) of the flow measurements on a subset of HCM patients with a calculated Qp:Qs ratio equal to or greater than 1.2. All statistical analyses were performed using MedCalc statistical software version 17.2 (MedCalc, Mariakerke, Belgium).

## Results

### Patient selection and baseline characteristics

**Fig. 2:**
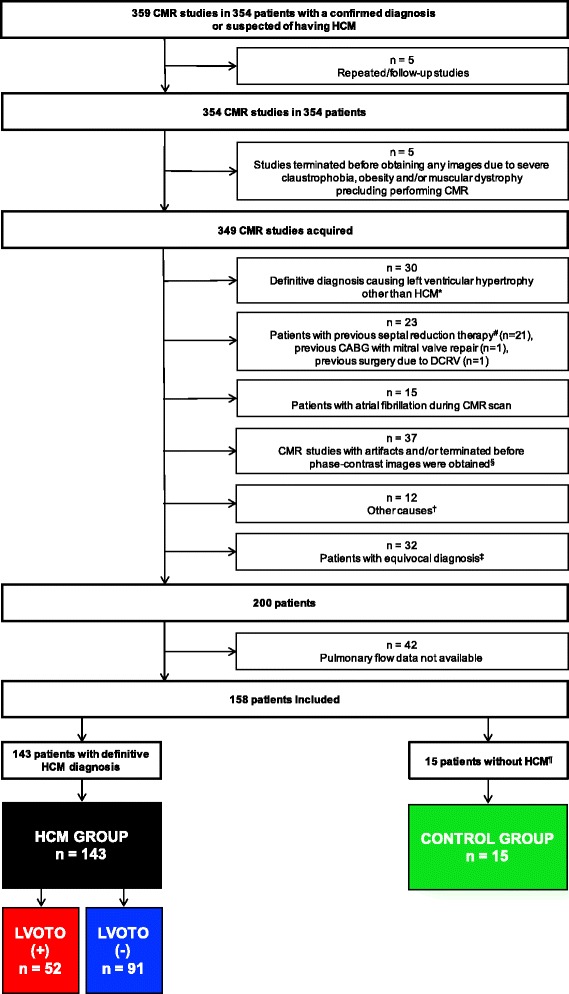
Flowchart outlining patient selection for the study. DCRV – double-chambered right ventricle, CABG – coronary artery bypass grafting. *A definitive diagnosis causing LV hypertrophy other than HCM or imitating HCM included the following: cardiac amyloidosis, Fabry disease, aortic stenosis, eosinophilic syndrome, and previous myocardial infarction with LV wall thinning and LV hypertrophy of the viable myocardium. ^#^Included 13 patients after alcohol septal ablation (one patient with subsequent endocardial radiofrequency ablation of septal hypertrophy), one patient with previous percutaneous transluminal septal coil embolization, and seven patients after surgical myectomy (three patients had previous alcohol septal ablation). ^§^Artifacts due to frequent premature ventricular or supraventricular contractions, claustrophobia, anxiety, post-stroke aphasia, and vertebral column stabilization implants. ^†^Included a history of atrial septal defect closure/intraatrial septum shunt, third-degree atrioventricular block, presence of left ventricular thrombus, and a significant amount of pericardial effusion/history of pericarditis. ^‡^Included patients with coexistence of HCM and severe hypertension; patients with papillary muscle abnormalities and/or prominent myocardial crypts without overt LV hypertrophy; and a proband or family member with borderline LV wall thickness that required further genetic testing. ^¶^Included family members of a patient with HCM studied as part of a screening program, patients with no LV hypertrophy present with a diagnosis of overestimation of ventricular wall measurements with the use of echocardiography, and patients with athlete’s heart

A total of 359 CMR studies of 354 patients with either an unequivocal diagnosis of HCM or suspicion of the disease were performed during the analyzed period. In cases of repeated studies, only the initial study was included. Studies terminated prematurely due to claustrophobia precluding the analysis of ventricular volumes or PC data were excluded. There were 122 patients excluded due to prespecified reasons (Fig. 2). The remaining 232 individuals included 180 patients with a definitive diagnosis of HCM, 32 patients with an equivocal diagnosis, and 20 patients without HCM. Pulmonary flow data were available in 143 HCM patients (79.4%) and in 15 individuals without HCM (75.0%) – these groups formed the final study population. A flowchart outlining the patient selection procedure for this study is shown in Fig. 2. The group without HCM served as a control group for the HCM patients.

None of the patients had more than trivial/mild aortic or pulmonary regurgitation defined as PC-derived regurgitation fraction ≤ 10%. Baseline characteristics of the study subjects are presented in Table 1. There were no differences in heart rate between the studied groups in either the cine or PC data. Additionally, no differences were observed in heart rate during the acquisition of Ao or MPA PC images (*p* = 0.61 for obstructive HCM patients, *p* = 0.07 for non-obstructive ones, and *p* = 0.58 for controls). Specifically, there were no differences in heart rate between aortic and pulmonary flow data in HCM patients with a Qp:Qs ratio ≥ 1.2 (*n* = 14). As shown in Table 1, there were statistically significant but small, clinically unimportant (ca. 2–3 beats per minute) differences in heart rate between the cine and PC data within the studied groups of HCM patients. There were no differences in Ao (*p* = 0.56), MPA (*p* = 0.86), or the Qp:Qs ratio (*p* = 0.61) calculated with PC data that were either uncorrected or corrected for background offset errors. Particularly, no significant differences were observed in HCM patients with a calculated Qp:Qs ratio ≥ 1.2 (*p* = 0.62, *p* = 0.58, and *p* = 0.71 for Ao, MPA, and Qp:Qs, respectively). In the same subsample of participants, high intra- and interobserver reproducibility of both Ao (ICC = 0.99 and ICC = 0.97, respectively) and MPA (ICC = 0.99 and ICC = 0.99, respectively) flow measurements was demonstrated. Representative curves of aortic and pulmonary flow in non-obstructive and obstructive HCM patients are shown (see Additional file 1: Figure S1).

**Table 1:** Baseline characteristics of HCM patients and controls

	HCM patients with LVOT obstruction (LVOTO+) *n* = 52	HCM patients without LVOT obstruction (LVOTO−) *n* = 91	Controls *n* = 15	Overall *p*-value	*p*-values for pairwise comparisons		
					LVOTO+ vs. LVOTO−	LVOTO+ vs. controls	LVOTO− vs. controls
Age, years	52.9 ± 13.7	44.6 ± 15.3	37.5 ± 17.8	<0.001	0.001	0.0007	0.11
Sex (males/females), n (%)	32/20 (61.5/38.5)	56/35 (61.5/38.5)	12/3 (80/20)	0.37	1.0	0.23	0.25
LVEDV (ml/m^2^)	97.1 (87.1–106.1)	90.7 (80.1–98.4)	95.8 (83.2–102.9)	0.005	0.0015	0.40	0.21
LVESV (ml/m^2^)	32.4 (27.5–39.1)	33.0 (28.0–38.6)	30.8 (29.2–40.9)	0.97	0.81	0.89	0.89
LVSV (ml/m^2^)	70.0 (58.0–70.6)	55.6 (47.3–60.1)	57.7 (54.6–66.8)	<0.0001	<0.0001	0.12	0.07
LVM (g/m^2^)	96.6 (74.1–109.7)	69.2 (59.1–86.2)	58.7 (50.1–66.2)	<0.0001	<0.0001	<0.0001	0.002
LVEF (%)	65.7 (61.7–69.9)	63.1 (59.4–66.0)	64.0 (61.1–69.0)	0.004	0.0009	0.23	0.36
Ao (ml/beat)	80.1 ± 19.6	82.2 ± 16.7	101.0 ± 17.1	<0.001	0.66	0.0006	0.0001
MPA (ml/beat)	90.4 ± 21.7	82.4 ± 16.5	101.1 ± 17.5	<0.001	0.014	0.08	0.0001
NetAo (ml/beat)	78.0 ± 19.6	80.8 ± 16.4	100.0 ± 17.1	<0.001	0.37	0.0001	0.0001
NetMPA (ml/beat)	89.6 ± 22.0	81.4 ± 16.3	100.1 ± 17.0	<0.001	0.012	0.10	0.0001
Qp:Qs	1.13 (1.05–1.21)	1.01 (0.99–1.03)	1.00 (0.99–1.01)	<0.001	<0.0001	<0.0001	0.11
Velocity encoding (cm/s)							
Ao PC images	160 (130–180)	140 (130–160)	130 (130–160)	0.0009	0.003	0.08	0.90
MPA PC image	120 (100–130)	100 (100–120)	120 (100–130)	0.003	0.001	0.68	0.10
Heart rate (beats per minute)							
Cine images	64 (58–69)^*^	66 (60–74)^#^	64 (58–69)^†^	0.28	0.18	0.25	0.50
Ao PC images	61 (56–69)^*^	64 (58–70)^#^	63 (61–76)^†^	0.22	0.13	0.17	0.61
MPA PC images	61 (57–68)^*^	63 (58–69)^#^	65 (60–72)^†^	0.25	0.29	0.12	0.30

Data are presented as the means ± SD, as medians with interquartile ranges or as numbers and percentages

LV parameters (LVEDV, LVESV, LVSV, LVM, and LVEF) were calculated using a method that included papillary muscles and trabeculations in the blood pool

*Ao* aortic, *LVEDV* left ventricular end-diastolic volume, *LVEF* left ventricular ejection fraction, *LVESV* left ventricular end-systolic volume, *LVOTO* left ventricular outflow tract obstruction, *LVSV* left ventricular stroke volume, *MPA* main pulmonary artery, *PC* phase-contrast

^*^ *p* = 0.006 and *p* = 0.003 for differences between cine vs. Ao PC data and between cine vs. MPA PC data, respectively

^#^ *p* = 0.0004 and *p* < 0.0001 for differences between cine vs. Ao PC data and between cine vs. MPA PC data, respectively

^†^ *p* = 0.07 and *p* = 0.63 for differences between cine vs. Ao PC data and between cine vs. MPA PC data, respectively

### Quantification of mitral regurgitation using either aortic or pulmonary flow

The Friedman test revealed significant differences between the MRvol quantification methods used (*p* < 0.00001 for all methods). Subsequently, pairwise comparisons were performed to explore the differences between the methods in detail.

**Fig. 3:**
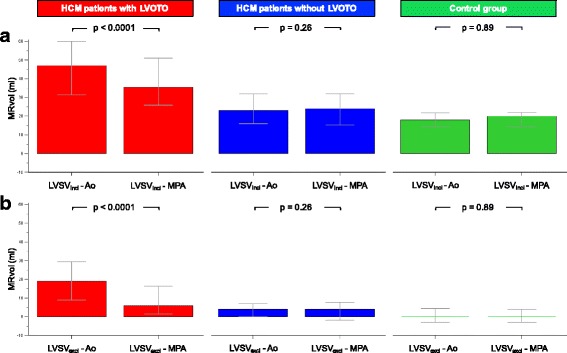
Differences in Ao-based and MPA-derived MRvol in three groups: HCM patients with LVOT obstruction (LVOTO), HCM patients without LVOT obstruction, and control subjects. **a** Upper row: Differences calculated using LVSV_incl_ (papillary muscles and trabeculations were included in the blood pool). **b** Lower row: Differences calculated using LVSV_excl_ (papillary muscles and trabeculations were excluded from the blood pool). Bars represent medians, and error bars represent interquartile ranges

First, we compared the quantification of MRvol as the difference between LVSV and aortic or pulmonary flow (Method 1 and Method 2, Fig. 1). Median MRvol_Aoi_ was higher than MRvol_MPAi_ in HCM patients with LVOT obstruction (47.0 ml, IQR = 31.5–60.0 ml vs. 35.5 ml, IQR = 26.0–51.0 ml; *p* < 0.0001) but not in non-obstructive HCM patients (23.0 ml, IQR = 16.0–32.0 ml vs. 24.0 ml, IQR = 15.3–32.0 ml; *p* = 0.26) or controls (18.0 ml, IQR = 14.3–21.8 ml vs. 20.0 ml, IQR = 14.3–22.0 ml; *p* = 0.89; Fig. 3).

**Fig. 4:**
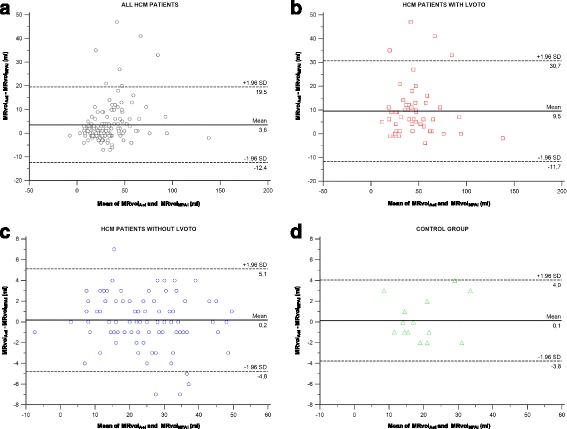
Agreement between aortic flow-based and pulmonary flow-derived MRvol (MRvol_Aoi_ vs. MRvol_MPAi_) in four groups. **a** All HCM patients (black circles). **b** HCM patients with LVOT obstruction (red squares). **c** HCM patients without LVOT obstruction (blue circles). **d** Control subjects (green triangles). The Bland-Altman plots demonstrating agreement are shown. The solid line indicates the mean of the differences between two parameters (bias). The dashed lines indicate the upper and lower limits of agreement (mean ± 1.96 SD)

To further investigate this issue, we performed Bland-Altman analyses to assess bias in aortic flow-based vs. pulmonary flow-based MRvol (Fig. 4). No significant bias was observed in either controls (bias = 0.1 ml; limits of agreement: −3.8–4.0; the highest difference was 4 ml) or HCM patients without LVOT obstruction (mean difference between measurements of 0.2 ml; limits of agreement: −4.8–5.1; the highest difference was 7 ml). However, in HCM patients with LVOT obstruction, the aortic flow-based MRvol (MRvol_Aoi_) was higher than the pulmonary-based MRvol (MRvol_MPAi_) (bias = 9.5 ml; limits of agreement: −11.7–30.7 with a difference of 47 ml in the extreme case).

As expected based on the above results, there were significant differences in Qp:Qs ratios between all HCM patients and controls (1.02, IQR = 1.00–1.08, range 0.93–1.90 vs. 1.00, IQR = 0.99–1.02, range 0.97–1.03; *p* = 0.002). However, differences were not observed between non-obstructive HCM patients and controls (Table 1). On the other hand, there were even more pronounced differences between the subgroup of patients with obstructive HCM and the controls (1.13 vs. 1.00; *p* < 0.0001, Table 1). There was also a significant difference in the median Qp:Qs ratio between patients with obstructive HCM and HCM patients without LVOT obstruction (Table 1).

**Fig. 5:**
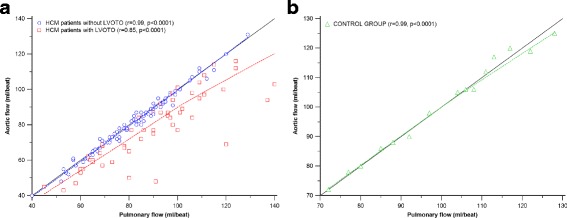
Correlations between net pulmonary flow and aortic flow. **a** In HCM patients with and without LVOT obstruction (LVOTO). **b** In the control group. *Black solid line* indicates agreement (equality line denoting perfect agreement between measurements). *Blue dashed line* indicates the trend line (correlation) in HCM patients without LVOTO (*blue circles*). *Red dashed line* indicates the trend line (correlation) in HCM patients with LVOTO (*red squares*). *Green dashed line* indicates the trend line (correlation) in control subjects (*green triangles*). For HCM patients without LVOT obstruction and for the control group, the trend (correlation) line almost fits the equality line, confirming almost perfect agreement between aortic and pulmonary flow. For HCM patients with LVOT obstruction, overestimation of aortic flow compared to pulmonary flow (demonstrated as the discrepancy between the agreement and trend lines) was observed

Subsequently, we analyzed the concordance correlation coefficients between net aortic and pulmonary flow in the study groups (Fig. 5). In both HCM patients without LVOT obstruction and controls, the concordance correlation coefficients exceeded 0.99 [0.9901, 95% confidence interval (CI) = 0.9850–0.9934, and 0.9931, 95% CI = 0.9798–0.9977, respectively], indicating almost perfect agreement [25, 26]. The concordance correlation coefficient of patients with LVOT obstruction, however, was 0.733 (95% CI = 0.607–0.824), indicating poor agreement [25, 26]. Additionally, the Pearson correlation coefficients for the associations between net aortic and pulmonary flow were calculated for each group (non-obstructive HCM patients: *r* = 0.99, *p* < 0.0001; obstructive HCM patients: *r* = 0.85, *p* < 0.0001; control subjects: *r* = 0.99, *p* < 0.0001; Fig. 5). Correlation coefficients between net aortic and pulmonary flow for obstructive HCM patients were lower than those for non-obstructive HCM patients (*p* < 0.0001) and controls (*p* < 0.0001).

### Quantification of mitral regurgitation using different LV segmentation methods

Next, we assessed similar associations using an alternative method of ventricular segmentation, namely, with the exclusion of papillary muscles and trabeculae from the blood pool and the inclusion of them in the LV mass (LVM) (Methods 3 and 4; Fig. 1). This resulted in higher LVEF and LVM values and lower LVEDV, LVESV, and LVSV values (Table 2).

**Table 2:** Comparison of absolute (not body surface area-indexed) left ventricular parameters assessed by two ventricular segmentation methods

	HCM patients (*n* = 143)			Control subjects (*n* = 15)		
	Trabeculations and papillary muscles included in the blood pool (_incl_)	Trabeculations and papillary muscles excluded from the blood pool (_excl_)	*p*	Trabeculations and papillary muscles included in the blood pool (_incl_)	Trabeculations and papillary muscles excluded from the blood pool (_excl_)	*p*
LVEDV (ml)	182.0 (157.0–204.0)	122.0 (103.0–137.0)	< 0.0001	198.0 (160.2–210.5)	147.0 (118.2–164.0)	0.0001
LVESV (ml)	64.0 (52.0–79.0)	27.0 (21.0–34.7)	< 0.0001	62.0 (53.7–82.2)	37.0 (27.5–49.7)	0.0001
LVSV (ml)	115.0 (97.3–129.0)	90.0 (78.0–105.0)	< 0.0001	119.0 (104.7–137.5)	103.0 (86.0–109.2)	0.0001
LVEF (%)	63.7 (60.1–67.5)	77.0 (72.0–82.0)	< 0.0001	64.0 (61.3–69.1)	73.0 (68.3–76.5)	0.0001
LVM (g)	152.0 (119.2–198.0)	214.0 (170.8–274.7)	< 0.0001	123.0 (95.5–141.2)	168.0 (138.0–199.2)	0.0001

Data are presented as medians with interquartile ranges

*EDV* end-diastolic volume, *EF* ejection fraction, *ESV* end-systolic volume, *LV* left ventricular, *LVM* left ventricular mass

**Fig. 6:**
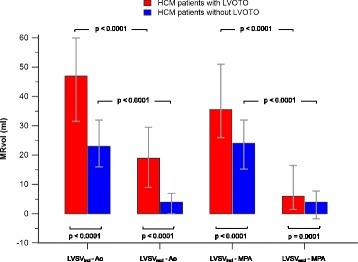
Comparison of MRvol values in HCM patients based on the method of LV segmentation. The groups were subdivided according to the presence (*red bars*) or absence (*blue bars*) of LVOT obstruction (LVOTO). MRvol values calculated as the differences between LVSV_excl_ and Ao or MPA were lower in all comparisons. Comparison of MRvol between patients with and without LVOT obstruction is also demonstrated. Independent of the method used for MRvol quantification, patients with LVOT obstruction exhibited higher MRvol values than non-obstructive HCM patients

As a consequence of the smaller LVSV_excl_ than LVSV_incl_, a smaller MRvol was observed with the use of LVSV_excl_ for both aortic flow-based (MRvol_Aoe_) and pulmonary flow-based calculations (MRvol_MPAe_) than that observed when using LVSV_incl_. This finding held for the entire HCM cohort, for subgroups divided according to the presence or absence of LVOT obstruction (*p* < 0.0001 for all comparisons, Fig. 6) and for control subjects (MRvol_Aoi_ vs. MRvol_Aoe_: 17.5 ml (IQR = 14.0–23.0) vs. −0.5 ml (IQR = −3.0–3.5), *p* < 0.0001; MRvol_MPAi_ vs. MRvol_MPAe_: 20.0 ml (IQR = 14.0–22.0) vs. 0.0 ml (IQR = −3.0–4.0), *p* < 0.0001).

### Comparison of mitral regurgitation between HCM patients with LVOT obstruction and those without LVOT obstruction

Independent of the quantification method used, MRvol was higher in HCM patients with LVOT obstruction than in HCM patients without LVOT obstruction (Fig. 6). However, as noted above, MRvol quantified as the difference between either LVSV_incl_ or LVSV_excl_ and aortic flow was higher than that obtained in calculations using pulmonary flow.

### Impacts of different mitral regurgitation volume quantification methods on mitral regurgitation grading

#### Comparison of aortic-flow-based and pulmonary flow-based mitral regurgitation grades

Comparing Method 1 (LVSV_incl_ − Ao) and Method 2 (LVSV_incl_ − MPA), there was moderate agreement in patients with LVOT obstruction (kappa = 0.45, 95% CI = 0.28–0.63; Table 3) but very good agreement in non-obstructive HCM patients (kappa = 0.84, 95% CI = 0.74–0.93; Table 4). Similar results were observed when comparing methods that used LVSV_excl_ to calculate MRvol (Method 3 vs. Method 4): kappa = 0.50, 95% CI = 0.32–0.68 for patients with LVOT obstruction vs. kappa = 0.90, 95% CI = 0.72–1.0 for patients without LVOT obstruction.

**Table 3:** Comparison of mitral regurgitation severity in HCM patients with LVOT obstruction: aortic vs. pulmonary flow-based MRvol grades

	Aortic flow-based MRvol grades calculated as LVSV_incl_ − Ao					
	<15 ml	15–29 ml	30–45 ml	45–59 ml	≥ 60 ml	Total
Pulmonary flow-based MRvol grades calculated as LVSV_incl_ − MPA						
< 15 ml	1	1	1	0	0	3 (5.8%)
15–29 ml	0	8	5	0	1	14 (26.9%)
30–45 ml	0	1	7	10	0	18 (34.6%)
45–59 ml	0	0	0	5	3	8 (15.4%)
≥ 60 ml	0	0	0	0	9	9 (17.3%)
Total	1 (1.9%)	10 (19.2%)	13 (25.0%)	15 (28.8%)	13 (25.0%)	52

*LVSV* left ventricular stroke volume, *MPA* main pulmonary artery, *MRvol* mitral regurgitation volume

**Table 4:** Comparison of mitral regurgitation severity in HCM patients without LVOT obstruction: aortic vs. pulmonary flow-based MRvol grades

	Aortic flow-based MRvol grades calculated as LVSV_incl_ − Ao				
	< 15 ml	15–29 ml	30–45 ml	45–59 ml	Total
Pulmonary flow-based MRvol grades calculated as LVSV_incl_ − MPA					
< 15 ml	18	3	0	0	21 (23.1%)
15–29 ml	1	36	3	0	40 (44.0%)
30–45 ml	0	2	23	1	26 (28.6%)
45–59 ml	0	0	0	4	4 (4.4%)
Total	19 (20.9%)	41 (45.1%)	26 (28.6%)	5 (5.5%)	91

There were no patients with MRvol ≥ 60 ml in the non-obstructive HCM group

Similar results were also obtained when a single threshold was used with a dichotomous grading system (non-severe vs. severe mitral regurgitation). For MRvol < 30 ml compared to MRvol ≥ 30 ml in patients with LVOT obstruction, kappa = 0.62 (95% CI = 0.38–0.85); in patients without LVOT obstruction, kappa = 0.88 (95% CI = 0.77–0.98).

Among 41 patients with LVOT obstruction with MRvol ≥ 30 ml based on aortic flow calculations, there were 7 (7/41; 17.1%) patients with MRvol < 30 ml based on pulmonary flow calculations, including one patient with MRvol calculated as ≥ 60 ml (specifically, 65 ml) using LVSV_incl_ − Ao but only 18 ml using LVSV_incl_ − MPA and one patient with MRvol calculated as between 30 and 44 ml (37 ml) using LVSV_incl_ − Ao but only 2 ml using LVSV_incl_ − MPA. The total number of patients with the highest mitral regurgitation grade (≥ 60 ml) decreased from 13 (25.0%) to 9 (17.3%) when MPA was used in the equation for the calculation of MRvol instead of Ao (relative decrease of 30.8%, Table 3).

In patients with LVOT obstruction, the exclusion of papillary muscles and trabeculations from the blood pool increased the percentage of patients reclassified from grades with higher mitral regurgitation based on aortic flow into the lowest grade of mitral regurgitation (MRvol < 15 ml) based on pulmonary flow. A total of 24 patients (46.2% of the analyzed subgroup with LVOT obstruction) exhibited the lowest mitral regurgitation grade (< 15 ml) when the MRvol = LVSV_excl_ − Ao equation was used, whereas there was a 50% increase to 36 patients (69.2% of the analyzed subgroup with LVOT obstruction) when the MRvol = LVSV_excl_ − MPA equation was used.

#### Comparison of mitral regurgitation grades calculated with different LV segmentation methods (_incl_ vs. _excl_)

Consequently, we analyzed the concordance between LV segmentation algorithms in mitral regurgitation grading. No agreement was found in the entire HCM population for all comparisons of mitral regurgitation grading based on MRvol calculated with either LVSV_incl_ or LVSV_excl_: Method 1 vs. Method 3 with aortic flow used as the PC data (kappa = −0.002, 95% CI = −0.04–0.04) and Method 2 vs. Method 4 with pulmonary flow used as the PC data (kappa = 0.002, 95% CI = −0.04–0.04). For both methods (aortic flow- and pulmonary flow-based), the exclusion of papillary muscles and trabeculations from the blood pool increased the number of patients with MRvol < 15 ml: for Ao from 14.0% (*n* = 20) to 76.9% (*n* = 110, see Additional file 2: Table S1); and for MPA from 16.8% (*n* = 24) to 84.6% (*n* = 121, see Additional file 3: Table S2). In particular, the grade was changed to < 15 ml in 23.1% (*n* = 3) of the 13 patients with aortic flow-based MRvol ≥ 60 ml when papillary muscles and trabeculations were excluded from the blood pool (LVSV_excl_). The number of patients reclassified from grade ≥ 60 ml to grade < 15 ml was even higher (44.4%; four out of nine patients) using the pulmonary flow-based MRvol.

Considering dichotomous thresholding (< 30 ml vs. ≥ 30 ml), the proportion of patients with MRvol < 30 ml increased from 49.7% (71/143) when MRvol was calculated as the difference between LVSV_incl_ and Ao to 90.9% (*n* = 130) when MRvol was calculated as the difference between LVSV_excl_ and Ao. For MPA-based calculations, using LVSV_incl_ resulted in 54.6% (78/143) of patients with MRvol < 30 ml, which increased to 95.8% (*n* = 137) of patients when LVSV_excl_ was used to calculate MRvol.

Interestingly, taking into consideration the very recently defined threshold of ≥ 60 ml for severe secondary and primary mitral regurgitation [21] and using aortic flow-based calculations, 84.6% of patients (11 out of 13) would be reclassified from severe to non-severe mitral regurgitation by simply changing the LV segmentation method from LVSV_incl_ to LVSV_excl_. Using pulmonary flow-based calculations, 88.9% of patients (8 out of 9) would be reclassified (see Additional files 2 and 3: Tables S1 and S2).

## Discussion

The main findings of our study are as follows: 1) in HCM patients with LVOT obstruction, aortic flow was significantly lower than MPA flow and, consequently, MRvol calculated as the difference between LVSV and Ao was significantly higher than MRvol calculated as the difference between LVSV and MPA flow; 2) the exclusion of papillary muscles and trabeculations from the blood pool and the inclusion of them in the LV mass calculation led to significantly lower MRvol than when papillary muscles and trabeculations in the blood pool were included; and 3) using the grading system for the severity of mitral regurgitation based on MRvol, there was substantial discordance between aortic flow-based and pulmonary-flow-based MRvol estimates, and there was no consistency between mitral regurgitation quantification using different methods of LVSV calculation (LVSV_incl_ vs. LVSV_excl_).

The most common approach for mitral regurgitation quantification using CMR imaging is based on two parameters: LVSV and Ao (MRvol = LVSV − Ao). Changes in either of these parameters will affect calculated MRvol. We demonstrated that the second parameter of this equation (i.e., Ao) was significantly affected by the presence of LVOT obstrution, leading to underestimated measurements when compared to pulmonary flow-based measurements. This finding has important clinical implications, as the estimated MRvol and mitral regurgitation grades may be higher than the true values. Under normal conditions, net aortic flow and net pulmonary flow should be almost equal, with a slightly greater pulmonary flow (taking into consideration that coronary flow reduces the net aortic flow when measured distal to the sinuses of Valsalva), and the ratio of pulmonary to systemic flow (Qp:Qs) should be close to 1. These calculations performed using CMR data are based on PC images and depend on a variety of factors, including turbulent flow. Previous studies showed that both bicuspid aortic valve and aortic valve stenosis cause turbulent flow in the ascending aorta, leading to flow measurement errors [14, 15]. LVOT obstruction, which is frequently observed in patients with HCM, is another mechanism leading to exaggerated turbulence in the LVOT, which spreads upstream to the level of the aortic valve and ascending aorta [27, 28]. Thus, we hypothesized and provided data confirming that LVOT obstruction may result in altered ascending aorta flow, which was found to be lower than pulmonary flow. This finding has important clinical implications regarding discrepancies in MRvol values. In our study, the maximal difference between aortic and pulmonary forward flow reached 47 ml in the extreme case, which translated into a 47-ml overestimation of MRvol and a Qp:Qs ratio of 1.74. Furthermore, the highest Qp:Qs ratio (1.90) was observed in a patient with an aortic net flow that was 41 ml higher than the pulmonary net flow.

The first parameter of the equation mentioned above (MRvol = LVSV − Ao) that is used for the calculation of MRvol is LVSV, which can be calculated in two ways: with or without the inclusion of papillary muscles and trabeculations in the blood pool. There is no consensus regarding the optimal method of ventricular segmentation, although some authors have suggested the superiority of the detailed (_excl_) segmentation method [29, 30]. Discrepancies in LV parameters calculated with the inclusion or exclusion of papillary muscles and trabeculations from the blood volume have been previously demonstrated in various populations, including patients with HCM [30–39]. Our results are in line with those of previous studies demonstrating that the exclusion of papillary muscles from the blood pool decreases LVSV with a resultant MRvol smaller than that obtained with the inclusion of papillary muscles in the blood volume. MRvol calculated with the inclusion of the papillary muscles/trabeculae in the blood pool is particularly overestimated in patients with coarse papillary muscles and trabeculae, such as patients with Fabry disease [33]. Similarly, patients with HCM exhibit more prominent papillary muscles than healthy subjects [40]. Among the controls in our study, who had no more than mild mitral regurgitation as assessed using echocardiography, median MRvol calculated with LVSV_incl_ was approximately 20 ml (up to 35 ml in the extreme case); similarly, Han et al. [30] demonstrated an average MRvol value of 20 ml (and a maximal value of 40 ml) in HCM patients with no evidence of mitral regurgitation or trivial mitral regurgitation on echocardiography. Therefore, one may state that inclusion of papillary muscles in the blood pool results in overestimation of MRvol (approximately 20 ml) compared to the true MRvol value (absent or trivial). In contrast, MRvol values close to zero calculated by subtracting aortic or pulmonary flow from LVSV_excl_ are in agreement with the values of MRvol predicted intuitively in patients with up to mild mitral regurgitation. This result supports the notion that the approach with the exclusion of papillary muscles/trabeculations from the blood volume should be preferred for LVSV calculations.

Taken together, our results indicate that alterations in the two necessary elements of MRvol quantification must be taken into consideration when assessing mitral regurgitation in HCM patients. Mitral regurgitation severity in HCM patients is important in clinical decision-making processes. We demonstrated that MRvol differed substantially based on the method of quantification used and that the grading system based on MRvol showed significant discrepancies independent of the thresholds used. We tested the agreement between the expanded five-stage grading system (<15 ml, 15–29 ml, 30–44 ml, 45–59 ml, and ≥60 ml) and the simplified dichotomous categorization (non-severe vs. severe). Regardless of the grading scale used, discordance was observed between the quantification methods used to define the grades. Particularly, discrepancies in grading stages based on LVSV_incl_ vs. LVSV_excl_ were extremely prominent and resulted in the reclassification of up to 44.4% of patients in the ≥ 60 ml MRvol category to the < 15 ml MRvol category. The total number of patients with MRvol < 15 ml increased by more than 5-fold simply by changing the ventricular segmentation method (from LVSV_incl_ to LVSV_excl_), and for patients with MRvol < 30 ml, there was an approximately 2-fold increase. Additionally, the utilization of dichotomous categorization with the most recent threshold of 60 ml as a cut-off value for both primary (degenerative) and secondary (functional) severe mitral regurgitation resulted in the reclassification of a substantial percentage (almost 90%) of patients from severe to non-severe mitral regurgitation. These data explicitly demonstrate that the same thresholds should not be used with different methods of MRvol quantification when grading the severity of mitral regurgitation.

The differences in LV parameters between two ventricular segmentation methods may seem to be larger than those previously published [30–39]. This is attributable to several factors. First, several previous studies considered papillary muscles and trabeculations separately – thus some studies excluded from the blood pool only trabeculations and some excluded only papillary muscles. In contrast, we excluded both papillary muscles and trabeculations from the blood pool, which resulted in more pronounced differences. The study by Chuang et al. revealed similar findings as those of our study when papillary muscles and trabeculations were simultaneously excluded from the LV volume [32]. Additionally, the papillary muscle and trabeculation volume expressed as a fraction of LVEDV was similar to that in our study (0.23 ± 0.03 vs. 0.25 ± 0.05). The second possible reason is the inverse relationship of age with the size of papillary muscles and trabeculae, as previously shown [32]. In other words, older patients have less pronounced papillary muscles and trabeculations; consequently, the difference between segmentation methods is smaller (decays) in older patients than in younger patients. Our population was rather young compared to that of some other studies. Different approaches to small trabeculations may also be the source of discrepancies between studies, as trabeculations smaller than 1.5 mm were not considered in some studies [33, 36, 38, 39]. Finally, interindividual differences in the degree of trabeculation may exist even among healthy subjects, as demonstrated by Chuang et al. in a large, community-dwelling population [32]. Our control group was rather small, and the vast majority was male. Considering all the above-mentioned factors and the wide normal reference range (e.g., 50th percentile of 32.3 ml and 95th percentile of 45.6 ml) for trabeculation and papillary muscle volume in healthy men [32], it is possible that some studies included more heavily trabeculated individuals than other studies.

Some of the patients included in our study had small negative MRvol values. Although highly reproducible, both the PC flow measurements and LVSV pose some estimation errors, which may rarely (in cases with no mitral regurgitation) result in slightly negative MRvol values. Small negative MRvol values could also be due to small (ca. 2–3 beats per minute) differences in heart rate during the acquisition of cine and PC data. Finally, background phase errors cannot be entirely discounted, although we provided data that phase errors did not significantly affect the results. It has recently been shown that offset errors are not clinically relevant for the CMR scanner used at our center [41]. In a study by Meierhofer et al. [41], baseline offset correction resulted in no improvement of flow measurements based on PC images acquired using an Avanto scanner. Similarly, there were no significant differences in parameters calculated with or without correction in our study. However, both the population in the study by Meierhofer et al. [41] and the sample cohort in our study were rather small; therefore, these issues should be addressed in future studies.

There were significant differences in age among patients with LVOT obstruction vs. patients without LVOT obstruction and control subjects. However, the main intent of this study was not to compare Ao and MPA flow between different cohorts but to compare those parameters within the given cohort (i.e., within patients with obstructive/non-obstructive HCM or controls). Using MPA flow, we aimed to internally validate the reliability of Ao PC data in patients with turbulent flow due to LVOT obstruction. Considering the main aim of the study, namely, comparison of Ao and MPA flow within given subjects, age differences should not affect the results, since age similarly affects both aortic and pulmonary flow within the same patient. Nevertheless, we performed multivariate analyses to check whether the differences in MRvol among different cohorts (HCM with LVOT obstruction, HCM without LVOT obstruction, and control group) were attributable to baseline differences between groups. By entering the presence of LVOT obstruction, age, gender, and velocity encoding sensitivity into the regression models, we found that the presence of LVOT obstruction (but not age or other factors) was an independent predictor of MRvol (data not shown). Thus, differences in mitral regurgitation severity between obstructive and non-obstructive HCM patients were not explained by variations in age between study cohorts but by the presence of obstruction.

The degree of LVOT obstruction together with the degree of mitral regurgitation vary depending on preload, LV contractility, and afterload [3, 42]. Moreover, medications, alcohol intake, and prandial status affect the LVOT gradient. Previous studies have shown that fluctuations in the LVOT gradient are inherent characteristics of HCM patients that stem from the dynamic nature of the obstruction [43–45]. Not only day-to-day but also beat-to-beat and respiratory variability in the LVOT gradient have been demonstrated [43–45]. In our study, the mean time between CMR and echocardiography assessment was 34 days; thus, differences in loading conditions between these two time points cannot be excluded. We are aware that it is not possible to control for a myriad of factors that affect the LVOT gradient, but we have tried to control the factors that are controllable. None of the patients underwent invasive procedures or a change in medical treatment within this interval. Nevertheless, wide spontaneous fluctuations in the LVOT gradient make it almost impossible to accomplish the experiments at two different time points with the same degree of LVOT obstruction. Performing two studies (CMR and echocardiography) in 1 day or in succession may not solve this issue. This limitation is common among all studies on HCM patients.

In addition to the above-mentioned issues, our study has several other limitations that should be acknowledged. Muzzarelli et al. [14] showed that PC acquisitions performed at levels other than the level of the ascending aorta (at the level of the aortic valve or LVOT) may be the solution to inaccurate flow quantification in the ascending aorta. In HCM patients, measurements in the LVOT in the presence of dynamic obstruction would be challenging and possibly erroneous due to the complex flow pattern caused by LVOT obstruction. Vortex formations originating in the LVOT make measurements proximal to flow turbulence impossible. Moreover, measurements at the aortic valve in the case of subvalvular obstruction (LVOT obstruction) would also be prone to artifacts and errors. Finally, the impact of shorter echo times on aortic flow measurements was not tested. However, we aimed to test our hypothesis using standard real-life parameters. Considering the results of our study, further studies should be considered to determine a solution for resolving inaccurate Ao data caused by LVOT obstruction. Using PC sequences with shorter echo times might be one solution [15]. The potential utility of pulmonary flow in clinical decision-making in patients with mitral regurgitation should be confirmed or refuted in further studies. In particular, a comparison of pulmonary flow-based vs. aortic flow-based mitral regurgitation volumes calculated using flow measurements performed at different levels of the aorta is warranted.

## Conclusions

Turbulent flow in the ascending aorta caused by LVOT obstruction significantly affects the estimation of Ao flow, leading to underestimation of Ao flow and, consequently, to higher MRvol values compared to those measured by MPA flow. In patients with obstructive HCM, pulmonary flow measurements should be considered in addition to conventionally used aortic flow measurements to provide a more reliable estimate of MRvol. Moreover, particular care must be taken in assessing mitral regurgitation using different LV segmentation approaches– namely, with or without the inclusion of papillary muscles and trabeculae. Considering the large degree of variability in MRvol when different analysis techniques (aortic vs. pulmonary flow; inclusion vs. exclusion of papillary muscles and trabeculae in the blood pool) are used, consensus and recommendations on the preferred technique are desirable. Ongoing large, multicenter studies (such as the Hypertrophic Cardiomyopathy Registry and the Global Cardiovascular Magnetic Resonance Registry of the Society for Cardiovascular Magnetic Resonance) with prospective follow-up data should provide more evidence to help address these issues [46, 47].

## Additional files

Additional file 1: Figure S1. Representative curves of aortic (left) and pulmonary (right) flow in non-obstructive (upper row, blue lines) and obstructive (lower row, red lines) HCM patients. In a patient with obstructive HCM, early peak flow is seen, with a subsequent decrease caused by the outflow tract obstruction. (PDF 18 kb)

Additional file 2: Table S1. Discordance between aortic flow-based mitral regurgitation volume (MRvol) grades in all HCM patients. (DOCX 16 kb)

Additional file 3: Table S2. Discordance between pulmonary flow-based mitral regurgitation volume (MRvol) grades in all HCM patients. (DOCX 15 kb)

## Abbreviations

Ao: Aortic forward flow
CI: Confidence interval
CMR: Cardiovascular magnetic resonance
HCM: Hypertrophic cardiomyopathy
ICC: Intraclass correlation coefficient
IQR: Interquartile range
LV: Left ventricular/left ventricle
LVEDV: Left ventricular end-diastolic volume
LVEF: Left ventricular ejection fraction
LVESV: Left ventricular end-systolic volume
LVM: Left ventricular mass
LVOT: Left ventricular outflow tract
LVSV: Left ventricular stroke volume
LVSV_excl_: Left ventricular stroke volume calculated with the exclusion of papillary muscles and trabeculations from the blood pool
LVSV_incl_: Left ventricular stroke volume calculated with the inclusion of papillary muscles and trabeculations in the blood pool
MPA: Main pulmonary artery
MRvol: Mitral regurgitation volume
MRvol_Aoe_: Mitral regurgitation volume computed as the difference between left ventricular stroke volume calculated with the exclusion of papillary muscles/trabeculations from the blood pool (LVSV_excl_) and aortic forward flow (Ao)
MRvol_Aoi_: Mitral regurgitation volume computed as the difference between left ventricular stroke volume calculated with the inclusion of papillary muscles/trabeculations in the blood pool (LVSV_incl_) and aortic forward flow (Ao)
MRvol_MPAe_: Mitral regurgitation volume computed as the difference between left ventricular stroke volume calculated with the exclusion of papillary muscles/trabeculations from the blood pool (LVSV_excl_) and main pulmonary artery forward flow (MPA)
MRvol_MPAi_: Mitral regurgitation volume computed as the difference between left ventricular stroke volume calculated with the inclusion of papillary muscles/trabeculations in the blood pool (LVSV_incl_) and main pulmonary artery forward flow (MPA)
PC: Phase-contrast
Qp:Qs: Ratio of net pulmonary flow to net aortic flow
SD: Standard deviation
