# Supplementary material for: Quantification of mitral regurgitation in patients with hypertrophic cardiomyopathy using aortic and pulmonary flow data: impacts of left ventricular outflow tract obstruction and different left ventricular segmentation methods
Source: J Cardiovasc Magn Reson. 2017 Dec 21;19:105. doi: 10.1186/s12968-017-0417-8 (PMC5740710; doi:10.1186/s12968-017-0417-8)

HCM patient without LVOTO

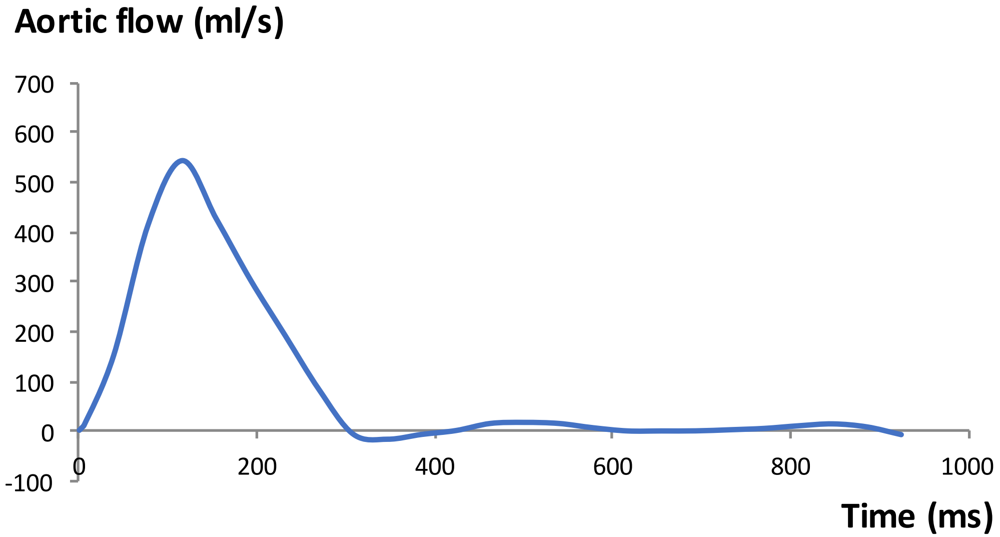

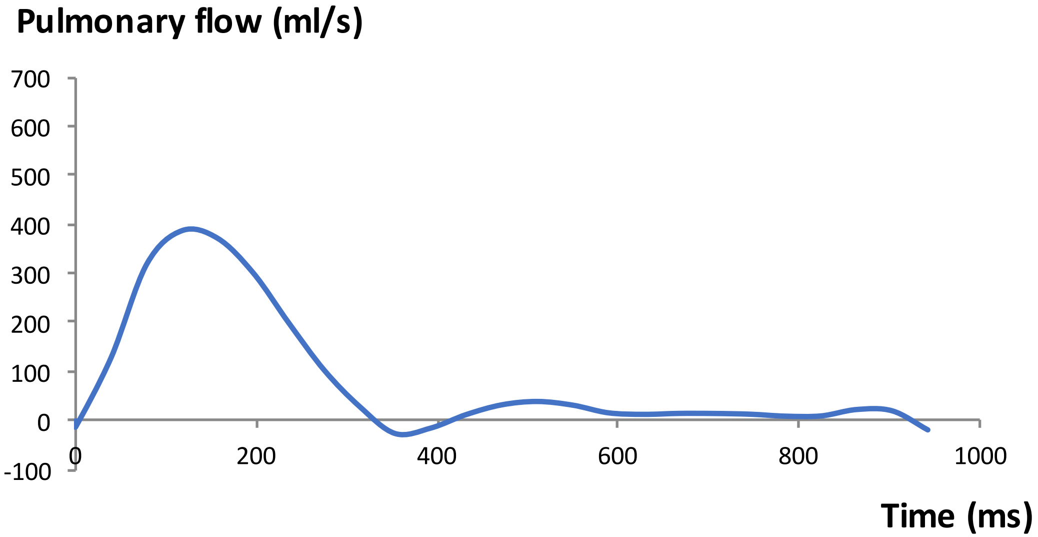

HCM patient with LVOTO

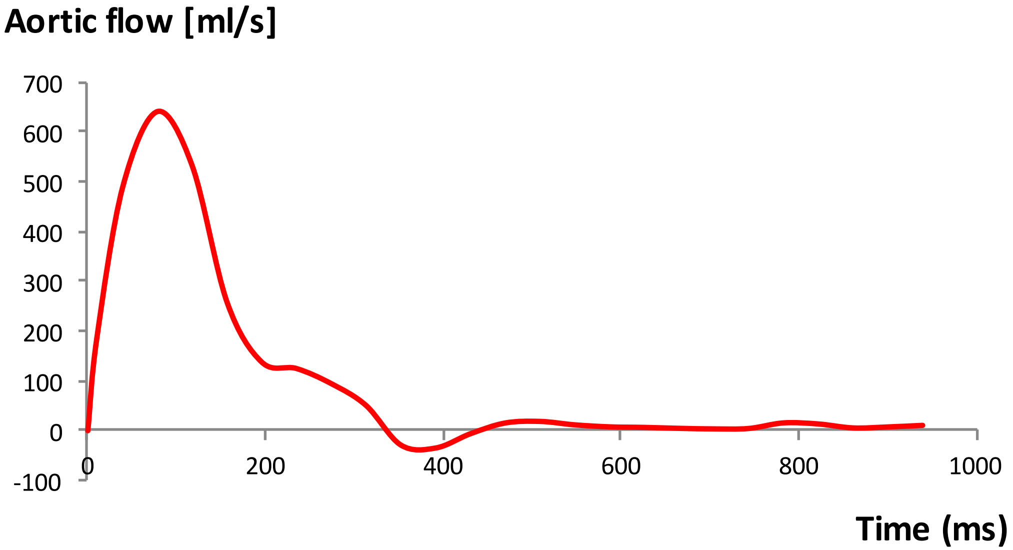

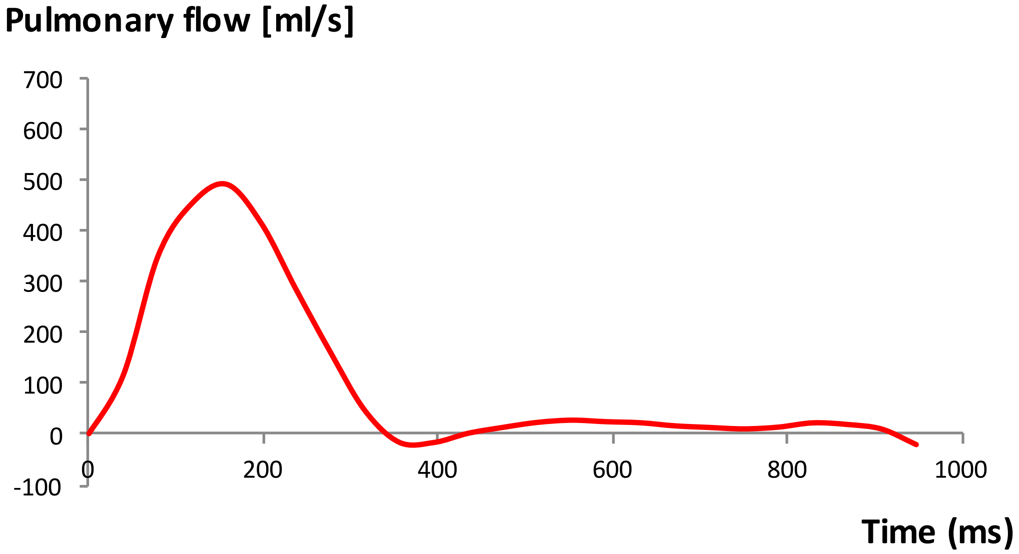

Supplement: Supplementary file 1 — Representative curves of aortic (left) and pulmonary (right) flow in non-obstructive (upper row, blue lines) and obstructive (lower row, red lines) HCM patients. In a patient with obstructive HCM, early peak flow is seen, with a subsequent decrease caused by the outflow tract obstruction. (PDF 18 kb) [file 12968_2017_417_MOESM1_ESM.pdf]
