# Supplementary material for: Quantification of mitral regurgitation in patients with hypertrophic cardiomyopathy using aortic and pulmonary flow data: impacts of left ventricular outflow tract obstruction and different left ventricular segmentation methods
Source: J Cardiovasc Magn Reson. 2017 Dec 21;19:105. doi: 10.1186/s12968-017-0417-8 (PMC5740710; doi:10.1186/s12968-017-0417-8)
Supplement: Supplementary file 3 — Discordance between pulmonary flow-based mitral regurgitation volume (MRvol) grades in all HCM patients. (DOCX 15 kb) [file 12968_2017_417_MOESM3_ESM.docx]

**Additional file 3: Table S2.** Discordance between pulmonary flow-based mitral regurgitation volume (MRvol) grades in all HCM patients

|  | Pulmonary flow-based MRvol grades  calculated as LVSV_excl_-MPA | | | | | |
| --- | --- | --- | --- | --- | --- | --- |
|  | <15 ml | 15-29 ml | 30-45 ml | 45-59 ml | ≥60 ml | TOTAL |
| Pulmonary  flow-based  MRvol grades  calculated as  LVSV_excl_-MPA |  |  |  |  |  |  |
| <15 ml | 24 | 51 | 34 | 8 | 4 | 121 (84.6%) |
| 15-29 ml | 0 | 3 | 10 | 3 | 0 | 16 (11.2%) |
| 30-45 ml | 0 | 0 | 0 | 1 | 3 | 4 (2.8%) |
| 45-59 ml | 0 | 0 | 0 | 0 | 1 | 1 (0.7%) |
| ≥60 ml | 0 | 0 | 0 | 0 | 1 | 1 (0.7%) |
| TOTAL | 24 (16.8%) | 54 (37.8%) | 44 (30.8%) | 12 (8.4%) | 9 (6.3%) | 143 |

MRvol was calculated as the difference between LVSV computed with two different ventricular segmentation algorithms [with either the inclusion (LVSV_incl_) or exclusion (LVSV_excl_) of papillary muscles and trabeculations from the blood pool and with their exclusion from ventricular mass calculations] and MPA.
